# "Hook"-calibration of GeneChip-microarrays: Chip characteristics and expression measures

**DOI:** 10.1186/1748-7188-3-11

**Published:** 2008-08-29

**Authors:** Hans Binder, Knut Krohn, Stephan Preibisch

**Affiliations:** 1 Interdisciplinary Centre for Bioinformatics, University of Leipzig, D-04107 Leipzig, Germany; 2 Interdisciplinary Center for Clinical Research, Medical Faculty; University of Leipzig, D-04107 Leipzig, Germany; 3 Max-Planck-Institute for Molecular Cell Biology and Genetics, D-01307 Dresden, Germany

## Abstract

**Background:**

Microarray experiments rely on several critical steps that may introduce biases and uncertainty in downstream analyses. These steps include mRNA sample extraction, amplification and labelling, hybridization, and scanning causing chip-specific systematic variations on the raw intensity level. Also the chosen array-type and the up-to-dateness of the genomic information probed on the chip affect the quality of the expression measures. In the accompanying publication we presented theory and algorithm of the so-called hook method which aims at correcting expression data for systematic biases using a series of new chip characteristics.

**Results:**

In this publication we summarize the essential chip characteristics provided by this method, analyze special benchmark experiments to estimate transcript related expression measures and illustrate the potency of the method to detect and to quantify the quality of a particular hybridization. It is shown that our single-chip approach provides expression measures responding linearly on changes of the transcript concentration over three orders of magnitude. In addition, the method calculates a detection call judging the relation between the signal and the detection limit of the particular measurement. The performance of the method in the context of different chip generations and probe set assignments is illustrated. The hook method characterizes the RNA-quality in terms of the 3'/5'-amplification bias and the sample-specific calling rate. We show that the proper judgement of these effects requires the disentanglement of non-specific and specific hybridization which, otherwise, can lead to misinterpretations of expression changes. The consequences of modifying probe/target interactions by either changing the labelling protocol or by substituting RNA by DNA targets are demonstrated.

**Conclusion:**

The single-chip based hook-method provides accurate expression estimates and chip-summary characteristics using the natural metrics given by the hybridization reaction with the potency to develop new standards for microarray quality control and calibration.

## 1. Background

DNA microarray technology enables conducting experiments that measure RNA-transcript abundance (so called gene expression or expression degree) on a large scale of genomic sequences. The quality of the measurement systematically depends on experimental factors such as the performance of the measuring "device", e.g., on the chosen array-type, the design of the chip-platform and -generation and on the particular probe design, on one hand; and also on the quality of the sample, e.g. on the source of RNA and the used hybridization-pipeline including the protocol of RNA-extraction, -amplification and -labelling, on the other hand. Other essential factors affecting the quality of the expression measures are the quality and up-to-dateness of the genomic information probed on the chip and last but not least, the performance of the calibration algorithm which transfers raw intensity data into suited measures of transcript abundance. This so-called calibration step aims at removing systematic biases from the raw data which, in the ideal case, would allow the determination of the exact number of transcript copies of every probed transcript and thus direct comparison of expression measures independently of the used array type and sample preparation protocol.

Apparent sources of variance can be, as for each experimental technique, divided into technical and biological ones, as well as, into systematic (see above) and random ones. The quality of the chip measurement and of the subsequent data calibration is characterized by their accuracy (the systematic bias between the measured and true expression value), precision (the uncertainty in replicated measurements), sensitivity (the expression range potentially covered by the measurement) and specificity (the selective power of the measurement to respond only to the specific targets).

The development of appropriate calibration method requires in the first instance appropriate models and metrics to identify, to assign and to quantify the biases in each measurement. In the accompanying paper we presented the basics of the so-called hook-method, a simple and intuitive approach providing a natural metric system to characterize the hybridization on a particular array. The method divides into two essential constituents: (i) the analysis of the data in terms of the competitive two-species Langmuir hybridization model using the so-called hook-plot and (ii) the correction of the raw intensities for parasitic effects such as the non-specific hybridization, saturation and sequence-specificity to output expression measures in intrinsic units which are defined by the properties of the measuring device. The hook method is a strict single-chip calibration approach which treats each array as an independent measurement. This way the method accounts for chip-specific systematic effects which the calibration step intents to correct.

In this paper we illustrate the performance of the hook method. We present examples dealing with different issues of array-measurements: the accuracy and precision of expression measures, the comparability of array experiments for different chip-generations, the effect of up-dating the probe assignments using latest genomic information, of RNA-quality and of different options of the preparation protocol such as labelling reagents and the type of the labelled molecule or replacing RNA-targets with DNA. We deliberately select a relatively wide range of different problems to illustrate the power of the method to estimate various systematic effects within a unique framework of chip-characteristic and to demonstrate the potential of developing new correction algorithms.

In the first part of the paper we summarize the essential chip characteristics provided by the hook-method. In the second part special benchmark experiments are analyzed to estimate transcript related expression measures. The third part deals with hybridization quality control based on the hook analysis.

## 2. Chip characteristics

### Hook parameters

**Figure 1:**
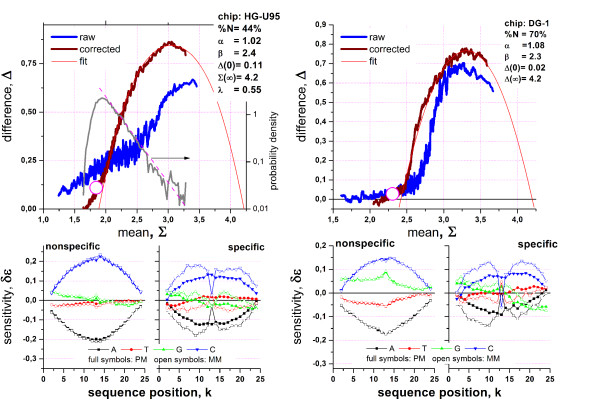
Hook-analysis of hybridizations on the human genome HG-U95 (left panel) and Drosophila genome DG-1 (right panel) GeneChips taken from the Genelogic dilution [1] and the GoldenSpike [2] experimental series: The upper panel shows the raw and the sensitivity-corrected hook curves, the fitted theoretical curve and the distribution of the Σ-signal values (right axis, only left panel). Each hybridization is characterized by the parameters given in the figure (see also Table 2). These chip-characteristics are obtained from the fit. They are related to the geometrical dimensions of the corrected hook curve (see text). The lower part in each panel shows the four sensitivity profiles: PM-N and MM-N (left) and PM-S and MM-S (right).

**Figure 2:**
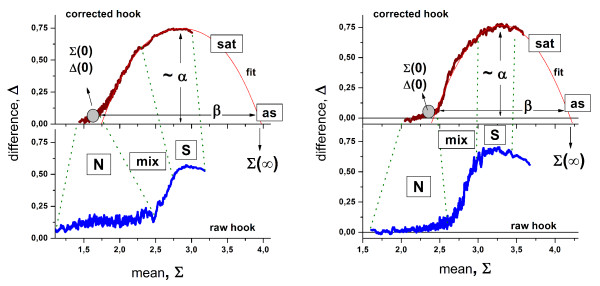
Hybridization ranges of the raw (lower part) and the corrected (upper part) hook-curves calculated from hybridizations of the HG-U95 (left) and DG-1 (right) Gene Chips (see also Figure 1). The dotted lines indicate the hybridization ranges characterized by predominantly non-specific (N) and specific (S) binding, by a mixture of significant S- and N-contributions (mix), by the progressive saturation of the probe spots with bound transcripts (sat) and by almost completely saturated probes (as). Affinity correction considerably changes the shape of the hook-curve and the extent of the hybridization ranges. The corrected hook-curve and the fit are characterized by their geometrical dimensions; width (β), height (~α), start- (Σ(0), Δ(0)) and end- (Σ(∞)) positions; which in turn characterize the particular hybridization in terms of the mean non-specific background contribution, the PM/MM-gain etc. (see Table 2 for details). Compare also with Figure 1: The HG-U95 data were taken from different experiment series (Affymetrix spiked-in series here [3] and Genelogic dilution series [1] in Figure 1).

Figure 1 depicts a typical graphical output-summary of the hook-analysis for two hybridizations performed on two different chip-types taken from the Genelogic dilution [1] and the GoldenSpike [2] experimental series (see also Figure 2 with data taken from the HG-U95 Latin square spiked-in series [3]). The Δ-vs-Σ plots characterize the hybridization of the particular chip. They are obtained by transforming the probe intensities of one GeneChip microarray into Δ = logI^PM ^- logI^MM ^and Σ = 0.5(logI^PM ^+ logI^MM^) coordinates and subsequent smoothing (I^PM ^and I^MM ^denote the spot intensities of the PM and MM probes after optical background correction; the logs are base 10 throughout the paper). The corrected version of the Δ-vs-Σ plot uses intensity values which are corrected for sequence-specific sensitivity effects. These plots are called hook-curves because of their typical shape. Additional characteristics of a particular chip-hybridization are the signal-density distribution and the four positional-dependent sensitivity profiles of the PM and MM probes upon specific and non-specific hybridization, respectively. These profiles are calculated from the intensity data of the chosen chip and used to correct the intensities for sequence-specific affinities.

The corrected hook-data are well fitted by the Langmuir-absorption model which predicts the theoretical curve shown in Figure 1. The fit provides characteristic parameters (see Table 1, see the accompanying paper [4] for details) of the particular hybridization judging properties such as the mean non-specific and specific signal, the saturation intensity and the mean PM/MM- gain of the sensitivity caused by the central mismatch of the MM probes (see Table 2, data are taken from the hook-analyses of more than 500 GeneChip arrays of different type and origin, see also [5] for details). Note that selected characteristics such as the non-specific binding strength (width) and the PM/MM-gain (height) are directly related to the geometrical dimensions of the hook-curve. Hence, the respective characteristics can be roughly and simply estimated by visual inspection of the Δ-vs-Σ plot.

**Table 1:** Geometrical parameters of the hook curve

**Hook parameter**	**symbol**	**typical range**	**characteristics**
**Start point**	Σ(0) ≈ Σ^*start*^,	1.0 – 2.5	Non-specific signal
	Δ(0) ≈ Δ^*start*^	0.0 – 0.15	PM/MM-gain (N)
**End point**	Σ(∞),	3.5 – 4.8	Saturation signal
	Δ(∞)	0	PM/MM-gain (as)
**Width**	*β *= Σ(∞)-Σ(0)	2.2 – 3.2	Measuring range, non- specific binding strength in logarithmic scale
**asymptotic height**	*α*	0.75 – 1.1	PM/MM-gain (S)
**decay constant**	*λ*	0.5 – 1.5	Decay rate of the density distribution of the Σ-values; this S/N-index characterizes the mean ratio of specific and non- specific binding (S/N- ratio) in the logarithmic scale.
**Expression index**	*φ *= (*β *- $\frac{1}{2}$Δ(0)) - *λ *≈ *β *- *λ*	1.5 – 2.5	Mean specific signal in logarithmic scale

**Table 2:** Overview of the hybridization characteristics extracted from the hook-analysis.

**Characteristics**	**Equation^b)^**	**characterizes...^b)^**	**Typical range^c)^**
***Chip-level ***(index "c" is omitted)			
Optical background, O^a)^	log *O *= ⟨log *O*⟩_*zones*_	...residual background intensity not related to hybridization; it is obtained using the Affy-zone algorithm performed prior to hook analysis	1.4 – 2.0
N-background signal^a)^	log *N *= Σ(0) + $\frac{1}{2}$Δ(0)	... mean background PM-intensity due to N-hybridization	1.0 – 2.5
PM/MM-gain in the N- range	log *n *= Δ(0)	...the PM-over-MM excess of the intensity presumably due to a certain amount of weakly (S-) expressed transcripts in the N-range	0.0 – 0.15
Saturation signal^a)^	log *M *= Σ(∞)	... the maximum possible intensity of the spots	4.0 – 4.9
N-binding strength^a)^	log *X*^*N *^≡ log *X*^*PM*, *N *^= -*β *+ $\frac{1}{2}$Δ(0)	... the (binding) strength of non- specific hybridization; measuring range of the chip	2.2 – 3.2
PM/MM-gain (S, the PM- over-MM excess of the intensity in the S-range)	log *s *= *α *- Δ(0)	...the effect of the mismatch on specific binding	0.8 – 1.1
Mean S/N-ratio^a)^	⟨*λ*⟩ = ⟨log(*R *+ 1)⟩_*R *> 0.5_	...mean (log-) S/N-ratio; R-range over which the density of expression values decays by one order of magnitude	0.2 – 1.5
Mean expression level^a)^	⟨*φ*⟩ = ⟨*λ*⟩ + log *X*^*N*^ ⟨*S*⟩ = 10^-⟨*φ*⟩^	...mean (log-) expression index in units of the specific binding strength	1.0 – 2.5
Standard deviation of the N- distribution^a)^	*σ*	...residual scatter of the corrected PM-intensities in the N-range (log- scale)	0.25 – 0.35
Percent non-specific, %N; fraction of N-probes	%N, f^absent ^= %N/100	Percentage of probe sets in the N- range;...amount of "absent" probes	20 – 95%

***Probe-set level ***(index "set" is omitted)			
Hook coordinates	Σ^hook^, Δ^hook^	...log-mean and log difference of the PM and MM intensities after optical background correction	1 – 4.7 and 0.0 – 1.1
S/N-ratio	R	...ratio of the specific binding strength of the probe set and the mean non-specific binding strength of the chip, signal-to-noise level	0 – 100, R = 0 indicates "absent" probes
expression level	L^S ^≡ L^PM, S^	...expression degree in intensity units (PMonly, MMonly and PM-MM estimates)	10 – 100,000
S-binding strength	X^S ^≡ X^PM, S^	...specific binding strength obtained as PMonly, MMonly or PM-MM_difference estimate	0 – 1

^a) ^characteristics refer to the PM-probes; for O, M and σ virtually equal values for PM and MM are obtained

^b) ^see the accompanying paper [4] for details

^c) ^ranges of typical values are taken from the hook-analyses of more than 500 GeneChip arrays of different type and origin (see [5])

Different parts of the hook have been assigned to (see Figure 2 from the left to the right) the N (non-specific)-, mix (mixed)-, S (specific)-, sat (saturation)- and as (asymptotic)- regimes of hybridization. These regimes reflect the fact that the contribution of specific hybridization to the spot intensities progressively increases along the rising part of the hook from tiny amounts in the N-regime to about 100% near the maximum. In contrast, the degree of saturation progressively increases along the decaying part from almost no saturation effects near the maximum to complete saturation in the as-regime. Note the considerable distortion of the N- and mix-regimes between the raw and corrected hooks. These marked differences between both hook-versions emphasize the importance of the correction step.

The N-range of the hook-curve is characterized by the variance of the underlying probe-level data, σ, which are well described by a normal distribution. The mean specific signal of the particular hybridization, <λ>, is calculated as log-mean of the S/N-ratio of the probe sets beyond a certain threshold (e.g. R > 0.5, see below). Note that the distribution of the specific signal is well approximated by an exponential decay in many cases. Then, the characteristic "decay" constant λ defines the Σ-range over which the probability of detecting a signal decays by one order of magnitude.

### Hook curves of different chip generations

**Figure 3:**
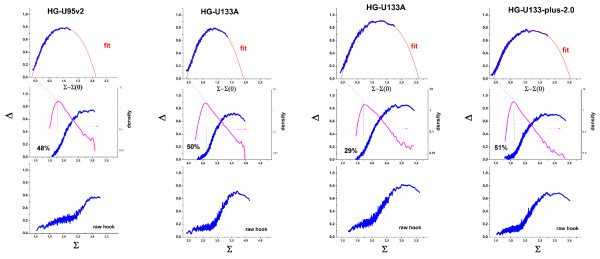
Hook-characteristics of GeneChips of different generations (see figure, from left to the right). The chips are hybridized with mRNA extracts from tumour samples (thyroid nodules, two parts on the left; [9] and references cited therein) and from the Universal Human Reference RNA (chips c and d; see [10] for details). The figures show the raw hook (below), the corrected hook (middle), the probability density distribution (middle, right axis) and the theoretical curve fitted of the mix-, S- and sat-ranges of the corrected hook curves (above). The percentage of absent probes (%N) is given within the figures.

Figure 3 shows a collection of representative hook-curves taken from four hybridizations of human-genome chips of different generations. Along the chip generations the spot-size of the probes decreases from 20 μm (U95), over 18 μm (U133A) to 11 μm (U133-plus2). The reduction of spot-size has enabled to increase the number of probe sets per chip from 16.000 over 22.000 to 54.000, respectively [6,7]. In addition, this development is accompanied by modifications of the reagent-kits and the scanning technique [7,8]. Importantly, also probe design and selection have been improved by applying more sophisticated genomic and thermodynamic criteria especially for chip generations following the U95. Chip data shown in Figure 3 refer to RNA prepared from tissue samples (thyroid nodules; [9]) and to Universal Human Reference RNA [10].

The different shapes of the uncorrected hook curves of the U95 and U133 chips, particularly the broader N-range of the former one, can be explained by the partially suboptimal quality of the probe selection for the U95-generation (which also applies to the design of the DG1-chip shown in Figure 1 and Figure 2) containing a relatively high number of weak-affinity probes. For the U133 series the N-range considerably narrows essentially due to better quality of the probes. It is important to note that our affinity correction levels out this difference to a large extent providing corrected hook curves of very similar shape for chips of different generations such as the U95 and U133 arrays.

We obtained analogous results for hundreds of GeneChip expression arrays of different specifications: chip generations, species (human, mouse, rat, drosophila, rice, arabidopsis etc.) and samples (patient cohorts, cell lines, benchmark experiments) [5]. Table 2 lists typical parameter-ranges obtained in these studies. For example, the PM/MM-affinity gain for specific hybridization shows that the central mismatch of the MM causes on the average the nearly tenfold (s ~ 7–11) increase of sensitivity of the PM-probes compared with that of the MM. On the contrary, for non-specific binding one expects on the average the same sensitivity for the PM- and MM-probes. The respective PM/MM-gain parameter however indicates a small but significantly increased PM-sensitivity, n ~ 1.05 – 1.25. We tentatively attribute this effect to false positive detections in the N-range, i.e. to a certain amount of specific hybridization among the absent probes (see below). The relatively narrow data-range of the obtained hybridization characteristics reflects the common physical-chemical basis of the method which is determined by properties such as the oligonucleotide density and size of the probe spots, the common MM probe-design and hybridization conditions. A particular example which demonstrates apparent inconsistencies between the expression estimates obtained from different chip-generations will be given below.

### Detection call

The onset and further increase of specific binding gives rise to a characteristic breakpoint of the hook curve which clearly separates the N- and mix- hybridization ranges. The corresponding change of the slope of the hook curve can be rationalized in terms of relatively strongly correlated PM- and MM-intensities in the N-range which progressively "decouple" upon increasing amount of specific binding because it much stronger affects the PM than the MM. We use the breakpoint to classify the probe sets into absent and present ones in analogy with the detection call provided by MAS5 [11].

To verify the used break-criterion in a simple illustrative fashion we analysed two special chip hybridizations. The GeneChip Yeast Genome 2.0 Array (YG 2.0) contains probe sets to detect transcripts of both, the two most commonly studied species of yeast, Saccharomyces cerevisiae and Schizosaccharomyces pombe. The YG 2.0 array thus includes 5,744 probe sets for 5,841 of the 5,845 genes present in S. cerevisiae and 5,021 probe sets for all 5,031 genes present in S. pombe. The evolutionary divergence between S. cerevisiae and S. pombe over 500 million years ago caused enough sequence divergence between the two species to require selection of separate probe sets for all genes, even the closest cross-species orthologs [12]. Due to this sequence divergence one expects only weak cross-species hybridization.

**Figure 4:**
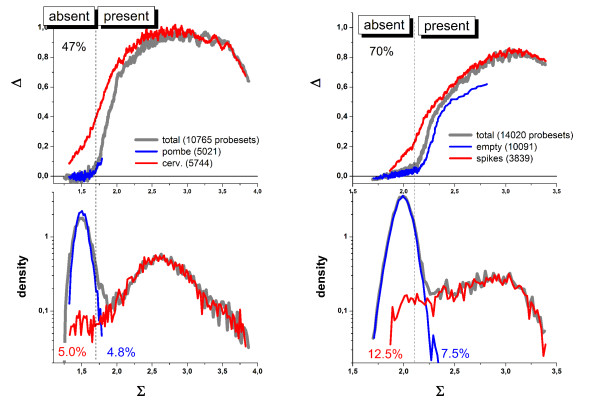
Present/absent characteristics of two hybridizations. **Left part**: The Yeast Genome 2.0 (YG 2.0) array contains about 50% probe sets designed for S. cerevisiae and S. pombe each. The hook refers to a chip hybridized with RNA taken from S. cerevisiae [13]. The hooks are calculated either for all probes or masking the probes of one of the two yeast species. The lower part shows the respective signal-density distributions. The added transcripts of S. cerevisiae give rise to virtually absent probes of S. pombe in the N-range of the hook curve. The relative amount of S. cerevisiae-probes called absent (red) and of S. pombe-probes called present (blue) are given within the figure. **Right part**: Hook curves for a DG1-chip taken from the Golden Spike series which has been hybridized with a definite collection of "spiked"-transcripts. The selective masking of the spikes and of the remaining "empty" probes shows that these probes accumulate in the S- and N-region, respectively. The relative amounts of empty probes called present and of spiked probes called absent are given in the figure.

Figure 4 shows the hook plot for a hybridization of the array with RNA from S. cerevisiae [13]. The break criterion provides a total absent rate of 47% which well agrees with the percentage of probe sets for S. pombe printed on the chip (~47%). Species-specific masking indicates that the absent probes originate nearly exclusively from the probe sets designed for S. pombe which indeed accumulate nearly completely in the N-range of the hook whereas the S. cerevisiae-probe sets cover the mix-, S- and sat-ranges as expected. About 5% of each fraction "overlap", i.e. they refer to present probe sets of S. pombe and absent sets of S. cerevisiae, respectively.

The second example was taken from the Golden Spike experiment in which PCR products from a Drosophila Gene Collection referring to 3,860 probes were spiked onto Drosgenome DG1-arrays [2]. On this array 10,131 probe sets out of the total number of 14,116 are called ,empty' because they are not assigned to any of the added cRNA spikes. Again the absent rate of 70% agrees with the fraction of empty probes (~72%). Selective masking of either the spiked or the empty probe sets shows that the latter ones indeed accumulate in the N-region and are called absent whereas the spikes are predominantly flagged as present (see right part in Figure 4).

The selective masking in these both examples shows that the simple break criterion gives rise to false present calls (of potentially absent probes) of less than 5 – 7% even if one neglects cross hybridization. The break-criterion provides a sort of detection limit for the specific expression signals. The detection call thus divides the probe sets into subsets with detectable and essentially not-detectable amounts of transcripts. The false present and false absent rates depend on the degree of cross hybridization and on other factors which will be addressed below.

In the next section we present other examples showing that the hook method reasonably estimates the detection limit of the particular array in terms of present and absent calls. The alternative calling-algorithm implemented in MAS5 calculates the so-called discrimination score (DS) of each probe pair which is directly related to its Δ-value [4,11]. Then, one-sided Wilcoxson's rank test is applied to the DS-values of each probe set together with appropriate threshold-settings to estimate whether the set is present or absent. The used test strongly penalizes negative PM-MM signal differences. More than 40% of all probe pairs amount to such "bright MM" (because MM > PM) in the N-range whereas its percentage steeply decreases with increasing Σ and virtually disappears in the S-range of the hook [14]. This trend explains the correlation between the call-rate obtained by both methods (see next section). For the examples presented here MAS5 provides a distinct smaller (36%) and an equal (70%) absent rate for the yeast and golden spike hybridizations, respectively.

On the other hand, the hook criterion includes both, the PM-MM difference in terms of the Δ coordinate and the mean total signal in terms of Σ. The latter value adds a second threshold which prevents probe sets with relatively strong mean signals to be called absent. Moreover, the break-criterion detects rather the change of the mutual correlation between the PM and MM signals caused by the onset of specific hybridization than a certain fixed signal level. As a result, the hook-criterion "dynamically" shifts with varying signal level using the break as a simple and reasonable landmark whereas the MAS5 threshold is statically and less intuitively given in terms of p-values typically predetermined by the default settings of the used analysis program.

## 3. RNA-expression

### Benchmark experiments with variable transcript concentration

**Figure 5:**
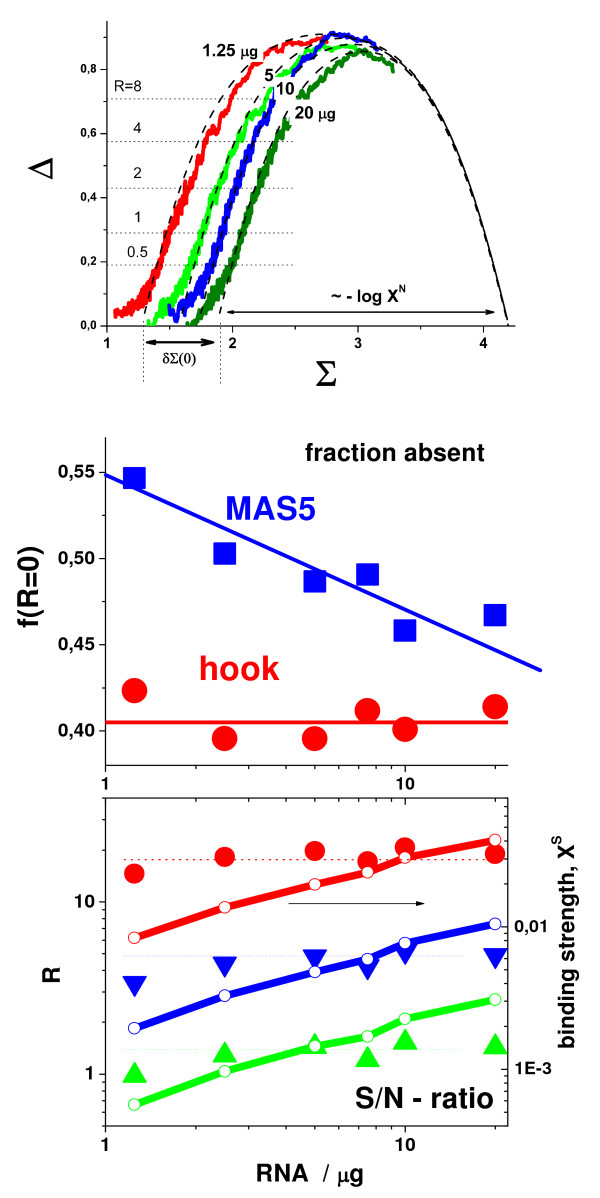
Genelogic dilution experiment: Hook curves for different dilution steps (upper panel), the fraction of absent probes (middle panel) and concentration measures (S/N-ratio and specific binding strength, lower panel) as a function of the amount of added RNA. The dilution of the hybridization solution shifts the increasing part of the hooks to the left and increases its width. The width is inversely related to the non-specific binding strength, ~-log X^N^, which consequently decreases upon dilution. The horizontal dotted lines in the upper part indicate the levels of different S/N-ratio (R); the dashed parabola-like curves are fits of the Langmuir-hybridization model. The hook method provides a virtually constant fraction of absent probes which corresponds to the essentially invariant S/N-ratio of the probes upon changing dilution. Contrarily, MAS5 provides an increasing fraction of absent probes (see middle panel). The lower part compares the S/N-ratio of selected probes which remain virtually constant upon dilution with the binding strength which progressively decreases (compare lines and solid symbols in the lower part; the diagonal lines refer to the right coordinate axis).

**Figure 6:**
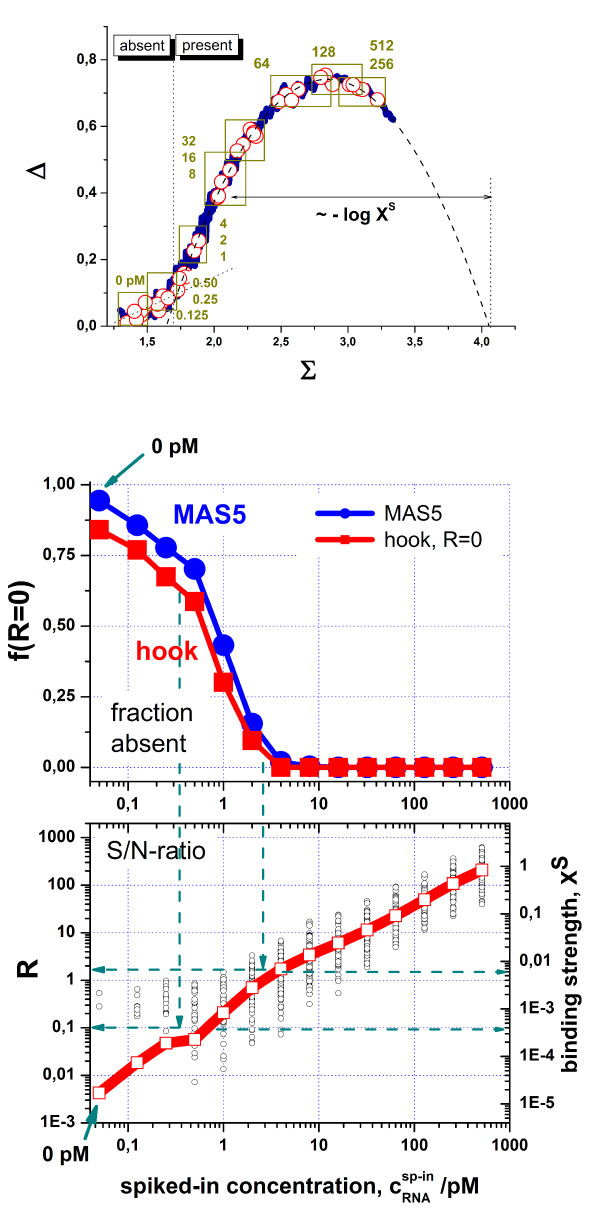
Affymetrix spiked-in experiment: The upper panel shows the hook obtained from one chip of this series. The predominant number of probes is hybridized with RNA of a HeLa-cell extract which was added to the chips to mimic a complex hybridization background (thick blue curve). The spike-probe sets are indicated by the open symbols and the respective transcript concentrations (see the numbers, the concentrations are given in units of pM). The horizontal distance between a spike position and the end point is related to the logarithm of the specific binding strength. The turning point between the N- and the mix-ranges defines the threshold for present probes. The dashed line is the fit of the Langmuir hybridization model to the data. The middle and lower parts show present/absent characteristics and the S/N-ratio of the spikes, respectively. The fraction of absent probes and the S/N ratio were calculated as mean values over all 42 chips of the experimental series (see thick lines). The open circles in the lower part show the individual probe-set values and thus the scatter of these points about their mean value. Spiked probes with nominal concentrations larger than 2 pM are "safely" called present. The S/N-ratio linearly correlates with the spiked-in concentration. The right axis of the lower part scales the expression estimates in units of the binding strength. The green dashed lines indicated that the threshold for calling probes as present corresponds to S/N-ratios R ≈ 0.1 – 2 and the S-binding strength of X^N ^≈ (0.5 – 5) 10^-3^.

Figure 5 and Figure 6 show the hook curves, the absent calls and concentration measures of two special benchmark experiments. In the GeneLogic dilution series, cRNA from human liver tissue was hybridized on HG-U95 GeneChips in various amounts [1]. The decrease of the degree of non-specific binding upon dilution widens the horizontal dimension of the hook curve (see upper panel in Figure 5). Dilution decreases the concentration of specific and non-specific transcripts in a parallel fashion leaving their concentration ratio virtually constant. As expected, the S/N-ratio R of selected probes remains essentially constant whereas the binding strength of specific binding progressively decreases (compare solid symbols and thick lines in the lower panel of Figure 5).

The hook-method provides a virtually constant fraction of absent probes independent of the dilution step (see middle part in Figure 5). This result can be rationalized in terms of the condition of R = const, which corresponds to virtually constant ordinate values, Δ ≈ const, in the mix-range of the hook-plot (see dotted horizontal lines in the upper panel in Figure 5). The horizontal shift of the hook upon dilution only weakly affects the fraction of probes below and above a certain R-value. Also the fraction of probes below and above the break criterion for classifying the probe sets into present and absent ones remains essentially constant. The virtually constant absent rate properly reflects the invariant composition of the hybridization solution. Contrarily, the fraction of absent calls estimated by MAS5 progressively increases upon dilution.

In the U133-spiked-in series of Affymetrix, a set of selected RNA-transcripts (the spikes) is added in definite concentrations to the hybridization solution [3]. The hybridization cocktail also contains a RNA-extract from HeLa-cells to mimic complex hybridization conditions. Figure 6 shows the typical hook-curve calculated from the intensity data of one chip of this experiment. The blue curve corresponds to the probe sets which are mainly hybridized with the non-spike RNA of the added background. The Δ-vs-Σ-coordinates of the probe sets detecting the spikes are shown by open circles. Their positions cover the full range of the hook curve and shift to the right with increasing transcript concentration (0 – 512 pM). Note that the distance of the position of a particular probe set relative to the end point is inversely related to the specific binding strength and thus to the specific transcript concentration.

Spike probe sets without specific transcripts (0 pM) and with transcripts of only tiny concentrations (< 0.5 pM) assemble mainly within the N-range of the hook curve. Figure 6 compares the absent call rates for the spikes obtained from the hook and MAS5 methods which both show similar results. The probability of flagging a probe absent increases upon decreasing transcript concentration. The absent rate thus reflects the resolution limit of the method for detecting small transcript concentrations. The vertical shift between the MAS5 and hook data can be adjusted by changing the threshold-parameters used in both methods.

The fit of the hook-equation provides the S/N-ratio R for each set of spiked-in probes which linearly correlates with the spiked in concentration (Figure 6, lower panel). The vertical axes in this figure show that the largest spike-concentration (512 pM) corresponds to a S/N-ratio of R≈ 200 (left axis) and to the specific binding strength of X^S ^≈ 1 (right axis). Comparison of the absent rates with the S/N-ratio indicates that the threshold for present calls refers to R ≈ 0.1 – 2 and to a binding strength for specific hybridization of X^N ^≈ (0.5 – 5) 10^-3 ^(see dashed arrows in Figure 6). Hence, the relevant measuring range of R and X^N ^covers about three orders of magnitude.

### Expression estimates

**Figure 7:**
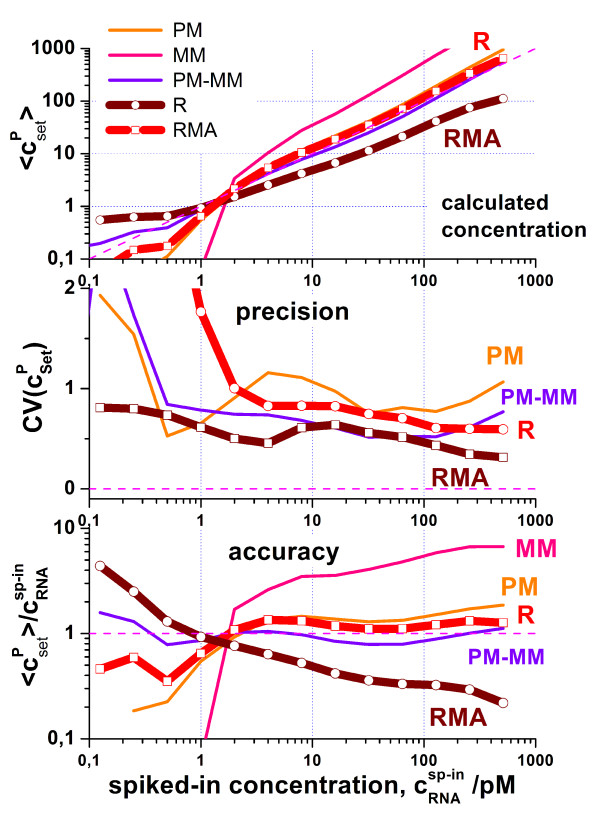
Expression estimates (upper panel, see figure for assignment), their coefficient of variation and the ratio of the estimated and the experimental ("true") spiked concentration (lower panel) as a function of the spiked concentration. The latter two measures estimate the precision and the accuracy of the expression values, respectively. The expression estimates in the upper panel are scaled to agree with the diagonal (dashed) line which refers to perfect results. The perfect precision and accuracy refer to zero (no scattering, middle part) and unity (lower part), respectively. All values are averaged over all probe sets detecting spiked transcripts. The figure compares the performance of the hook expression estimates (PMonly, MMonly, PM-MM and R) with that of RMA (see text).

The hook-methods provides potentially four alternative expression measures of each probe set: the S/N-ratio R, which is obtained from the direct fit of the transformed two-species Langmuir isotherm to the hook curve; and PMonly, MMonly and PM-MM-difference estimates which are calculated as the mean generalized logarithm of the background- and sensitivity corrected and de-saturated signal values averaged over the background distribution. The corrections for the latter three expression values are estimated from the hook-curve analysis. Figure 7 compares the performance, accuracy and precision of the different alternative measures in terms of their correlation with the known spiked-in concentration. The precision reflects the scattering of the estimated data about their mean and was therefore estimated as the respective coefficient of variation. The accuracy reflects the systematic deviation of the estimated from the spiked concentration. Hence, it was quantified as the ratio of the estimated concentration and the known concentration of the spikes. For sake of comparison we also show RMA (robust multiarray analysis, [15,16]) expression estimates in Figure 7.

It turns out that all considered methods except MMonly are comparably precise at larger transcript concentrations c^sp-in ^> 2 pM, at which the transcripts are safely called present (see previous paragraph). Note that the direct fit of the hook equation to the data provides the S/N-ratio which represents only a rough measure of the expression degree. The PMonly and PM-MM estimates more precisely correct the signals for the non-specific background contribution. It does therefore not surprise that these measures outperform the S/N-ratio R at smaller c^sp-in^-values in terms of precision. The MMonly expression values are by far the most imprecise ones which does not surprise because the specific signal level and thus the sensitivity of the MM-probe intensities are smaller by nearly one order of magnitude compared with the respective PMonly and PM-MM measures at a comparable non-specific background level. The coefficient of variation of the MMonly expression estimates exceeds CV > 2 over the whole concentration range which exceeds the maximum scaling used in Figure 7.

The hook-measures clearly outperform the RMA-values in terms of the accuracy of the expression values. Note that RMA uses a linear intensity approximation which ignores saturation at high transcript concentrations at one hand-side and corrects the intensities for non-specific hybridization using a global background level on the other hand-side. As a consequence, RMA systematically underestimates the change of the expression values especially at high and small transcript concentrations (see also [5] for a detailed discussion). Note that RMA represents a multichip- method which processes a series of chips to adjust the probe-specific sensitivities. In contrast, the hook method provides strictly single-chip estimates which are based on the intensity information of only one particular chip. The accuracy of the PM-MM estimates perform best among the methods at small transcript concentrations presumably because the explicit use of the MM intensities well corrects for sequence-specific background effects not considered by the positional dependent sensitivity model used by the hook method.

In this context we explicitly refer to the so-called effect of "bright" MM, i.e. a certain amount of about 40–50% of negative PM-MM intensity differences on each chip [17,18]. This systematic bias has been explained by the intrinsic purine-pyrimidine asymmetry of base pairings in the non-specific DNA/RNA probe/target duplexes [14,19,20]. The sensitivity correction used by the hook method explicitly corrects the raw intensity data for this sequence effect.

### Reproducibility across GeneChip-generations

Up to now a large number of microarray data has been collected in public repositories such as GEO (Gene expression Omnibus of NCBI) or ArrayExpress (EBI) referring to a wide variety of different conditions, specimen and array-types. One important challenge in microarray analysis is to take full advantage of these previously accumulated data, e.g., for combining different datasets to get a more comprehensive view in comparative analyses. Difficulties related to the heterogeneous character of array platforms, chip types and hybridization protocols in most cases hinder such meta-analyses. Consistencies and inconsistencies between chip platforms and -types have been previously addressed in a number of studies [21-25].

A recent study reports that even identically composed probe sets containing identical numbers and sequences of probes on different GeneChip-types can produce significantly different values of gene expression in cross-chip comparisons for samples containing the same target RNA [10]. Particularly, this study compares the newer HG-U133 plus 2.0 (P-chip) with the previous-generation HG-U133A (A-chip) array. The nearly 55.000 probe sets of the former chip integrate the more than 22.000 probe sets of the HG-U133A chip and, in addition, the probe sets of the HG-U133B array. In the study both, the A- and P-arrays were hybridized with the same Universal Human Reference RNA.

For subsequent comparison of the expression values the authors masked the additional probe sets on the P-chip ("not A"-probes) and processed only the common probe sets present on both chips ("A"-probes) using MAS5 and a combination of global and invariant-set normalizations (see ref. [10] for details). The analysis revealed a number of differentially expressed genes which is much larger than the number expected by chance despite the identical probes and target RNA.

**Figure 8:**
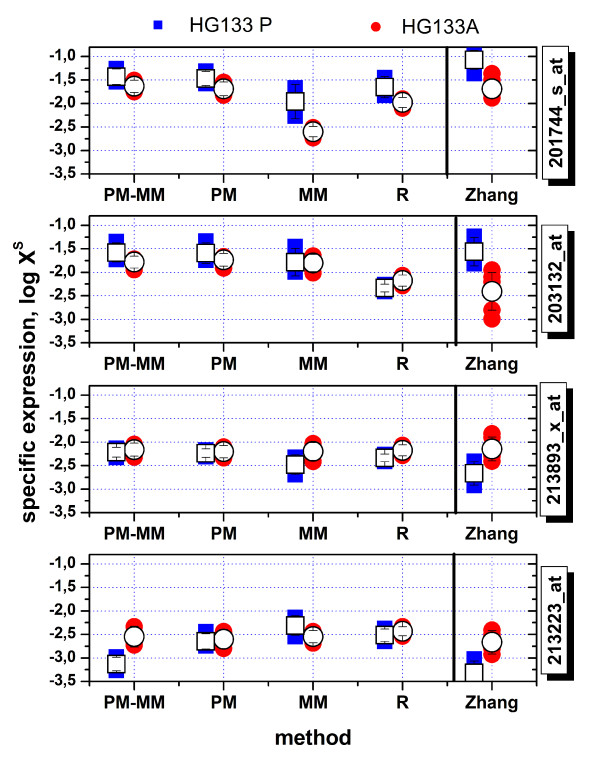
Cross-chip comparison of the expression estimates of four selected probe sets taken from the HG-U133A and HG-U133plus2 arrays (chip data were taken from [10]). Both chip types were hybridized with human reference RNA in five replicates (solid symbols). The open symbols are the log-means over the replicates. Expression measures taken from ref. [10] were compared with the four alternative measures provided by the hook-method. Note the systematic shift of the expression values between both different chip-types which changes sign upon increasing expression value. The chip-type specific bias considerably reduces for the hook-measures. The MMonly-method performes worst among the hook-methods. (see also Figure 7). The Zhang-measures are given in arbitrary units which were scaled for comparison with the hook data.

Figure 8 compares the expression values of four probe sets selected by Zhang et al. as representative examples ranging from small to high expression levels to illustrate the bias caused by the chip-types (see also Fig. 3 in ref. [10]). Note that the difference between the expression values of both chip-types inverses sign upon increasing expression suggesting that simple re-scaling of the data does not solve the problem.

We re-analyzed these chip-data using the hook-method. The left part of Figure 8 shows that the systematic difference between the chip-types essentially disappeared at small expression levels and it is clearly reduced compared with the data of Zhang et al. at larger expression levels. Parallel analyses which either consider or not consider the not A-probes provide virtually the same results (data not shown). We tentatively attribute this improvement to the sequence correction of the intensities and to the proper estimation of the non-specific background correction.

**Figure 9:**
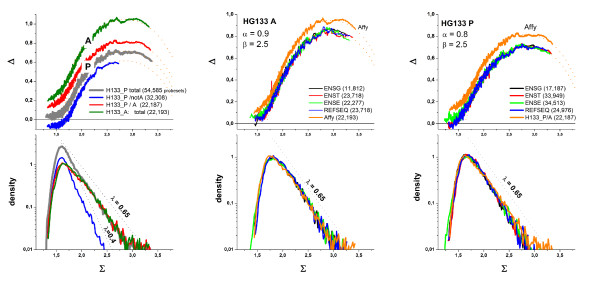
Signal distributions (below) and corrected hook curves (above) of Universal Human Reference RNA hybridized on HG133A and HG133plus2 GeneChips (raw data were taken from [10]). The probe sets are composed either according to Affymetrics default settings (left part and "Affy" in the other parts), or using different customized transcript definitions (see ref. [30]; version 10; middle and right part) based on the annotations of different resources: ENSEMBLE (ENSG, ENSE, ENST), REFSEQ (see text). The probe sets of the HG133plus2 array were split into two subgroups which are either represented on both chip-types ("A") or on the P-chip only ("notA"). See the legends within the figure. The respective number of probe sets per array is given within the parentheses. The dotted lines in the lower panel serve as guide for the eye to characterize the respective decay constants λ. The hooks in the left part and the "Affy" hooks in the other parts are shifted in vertical direction each to another for sake of clarity. The dotted curves in the upper panel are fits of the hook-equation. The essential parameters are given in Table 3.

In the next step we compare the hook-curves of the P- and A-chips to identify possible differences of their hybridization characteristics. Examples of raw and corrected hooks taken from this series are shown in Figure 3 (see the two parts on the right). In Figure 9 we re-plotted the corrected hooks and the density distributions for direct comparison. The characteristics of the P-chip were calculated using either all probes or the two subsets of probes shared (A-probe sets) and not-shared (not-A-probe sets) with the A-array. All hook versions fit well to the theoretical function. Table 3 summarizes the extracted parameter values.

**Table 3:** Hook characteristics of HG-U133A and HG-U133plus2 chips hybridized with the same RNA using different probe set definitions^a)^

**probe set definition**	**chip-type**	**Optical BG**	**non- specific BG**	**N-binding strength**	**PM/MM- gain (S)**	**PM/MM-gain (N)**	**mean S/N-index**	**mean expression index**	**percent absent**	**probe utilization**^d)^
		**logO**	**logN**	**β**	**α**	**logn**	**<λ>**	**<φ>**	**%N**	**%P** **# of probe sets**
**Affymetrix probe sets^b)^**										
**total**	HG- U133A	1.89 ± 0.04	1.71 ± 0.04	2.70 ± 0.04	0.99 ± 0.03	0.10 ± 0.01	0.61 ± 0.03	2.09 ± 0.05	34 ± 3	100% 22,193
**total**	HG- U133plus2	1.81 ± 0.04	1.65 ± 0.08	2.75 ± 0.07	0.85 ± 0.01	0.07 ± 0.01	0.57 ± 0.03	2.18 ± 0.07	50 ± 3	100% 54,585
**A**	HG- U133plus2	1.82 ± 0.06	1.63 ± 0.08	2.76 ± 0.07	0.86 ± 0.01	0.09 ± 0.01	0.65 ± 0.05	2.11 ± 0.07	29 ± 3	41% 22,187
**notA**	HG- U133plus2	1.80 ± 0.06	1.67 ± 0.09	∞	0.83 ± 0.01	0.06 ± 0.01	0.45 ± 0.03	x	64 ± 5	59% 32,308

**Customized probe sets^c)^**										
**Ensemble gene**	HG- U133A	1.89 ± 0.05	1.71 ± 0.03	2.77 ± 0.02	0.97 ± 0.03	0.12 ± 0.003	0.56 ± 0.03	2.21 ± 0.04	23 ± 3	68% 11,834
	HG- U133plus2	1.82 ± 0.06	1.61 ± 0.09	2.74 ± 0.07	0.84 ± 0.01	0.08 ± 0.02	0.56 ± 0.05	2.18 ± 0.07	21 ± 3	48% 17,215
**Ensemble transcript**	HG- U133A	1.88 ± 0.05	1.71 ± 0.03	2.67 ± 0.05	0.95 ± 0.03	0.10 ± 0.01	0.57 ± 0.03	2.10 ± 0.05	19 ± 1	71% 23,740
	HG- U133plus2	1.81 ± 0.06	1.65 ± 0.09	2.68 ± 0.07	0.79 ± 0.11	0.04 ± 0.09	0.57 ± 0.04	2.08 ± 0.07	18 ± 11	48% 33,977
**Ensemble Exon**	HG- U133A	1.88 ± 0.05	1.69 ± 0.04	2.64 ± 0.06	-0.97 ± 0.02	0.11 ± 0.01	0.58 ± 0.03	2.06 ± 0.06	23 ± 3	63% 22,299
	HG- U133plus2	1.81 ± 0.06	1.62 ± 0.08	2.72 ± 0.13	0.84 ± 0.01	0.09 ± 0.01	0.57 ± 0.06	2.15 ± 0.15	25 ± 5	43% 34,541
**Refseq**	HG- U133A	1.88 ± 0.05	1.68 ± 0.06	2.64 ± 0.07	0.96 ± 0.02	0.09 ± 0.02	0.60 ± 0.05	2.04 ± 0.07	17 ± 3	72% 17,531
	HG- U133plus2	1.83 ± 0.06	1.67 ± 0.08	2.65 ± 0.12	0.83 ± 0.01	0.09 ± 0.01	0.61 ± 0.05	2.04 ± 0.12	27 ± 7	48% 25,004

^a) ^Raw intensity data were taken from ref. [10]; human reference RNA has been hybridized onto both chip types in 5 replicates. The data are log-averages/±SE

^b) ^Probe set definition of the manufacturer; total...all probe sets; A/notA...probe sets shared/not shared between the P- and A-chips

^c) ^Customized probe sets were filtered using genomic information provided by Ensemble (gene, transcript or exon related) and Refsequ (see [30]); probe set definitions were downloaded from  (version 10) as CDF and probe-sequence files

^d) ^Percent and total number of the probes on the respective chip which are used in the respective analysis. Note that the number of probes per set varies between 4 and more than 30 for the customized sets. The data are taken from

The widths of the hooks and thus the respective level of non-specific binding are virtually the same for the P- and A-arrays. The not-A-probe sets are, on the average, distinctly less expressed than the A-probe sets as indicated by the more than twice as large amount of absent probes (%N = 64% versus 29%) and the smaller decay rate of the respective density distribution (λ = 0.45 versus 0.65). The percentage of absent probe sets on the P-chip (50%) represents the average of the respective contributions of A- and not-A-probes where the not-A-probes obviously add a considerable larger amount than the A-probes. The total density distribution of the P-chip well agrees with the distribution of the not-A-probes in the N-range and with that of the A-probes in the S- and sat-ranges. In summary, the hybridizations on both chips well agree in terms of the general target properties (N-background, decay rate) but differ with respect to the general probe characteristics (%N). The latter effect simply reflects the different probe-selections of the manufacturer for each chip type.

Besides these essentially common characteristics, the hook-analysis revealed one significant difference between the chip types, namely the significantly increased height parameter α for the A-chips. This parameter characterizes the PM/MM-gain of the specific signals, or, in other words, the mean incremental effect of introducing one central mismatch into specific probe/target duplexes. Here one expects however virtually identical α-values for the A- and P-chips because the mismatch design and the nominal probe length are identical for both array-types. On the other hand, subtle deviations from the nominal probe design owing to deficiencies of fabrication and/or variations of the hybridization conditions in different preparations can however affect the observed maximum PM/MM ratio: For example, the in-situ synthesis of the GeneChip probes usually produces a non-negligible fraction of truncated probe-oligomers not synthesized to full nominal length. This effect gives rise to systematic deviations from the Langmuir isotherm and, more importantly, it will affect the PM/MM-gain because the relative effect of one middle-mismatch is expected to increase with decreasing length of the probe oligomers [26,27]. Also the post-hybridization washing step upon chip preparation is expected to affect the apparent PM/MM-ratio and the binding law as well [28,29]. We suggest that subtle differences of the hybridization law due to details of chip-manufacturing and/or handling of the chips upon preparation as well as evolving instrumentation and instrument protocols give rise to slightly biased expression data between different array types and/or different batches of chips of the same type. The latter conclusion was derived from another chip series for which we observed a reversed relation of the PM/MM-gain, namely a larger value for the P-array compared with the A-array [5] (see also the two A-chips in Figure 3). Selected hook parameters can serve as indicators of such effects and can provide hints for their origin.

### Updated probe sets

One possible approach to partially level out chip-type specific differences is the matching of the probe sets of different array types using genomic sequence information updated with respect to the original probe set assignment of the manufacturer. Recent studies show that significant percentages of existing GeneChip probe set definitions are no longer consistent with gene and transcript assignments in actual versions of public databases. The probe identity issue is of critical importance, as it significantly affects the expression values summarized on probe set level and thus their interpretation and understanding [30,31]. Dai et al. [30] performed reanalysis of probe and probe set annotations resulting in publicity available, regularly updated probe set definitions for most of the GeneChip-types. A series of probe selection and grouping criteria utilizing the latest sequence and annotation information taken from databases such as REFSEQ or ENSEMBLE (gene, transcript and exon based) are applied. (i) This filtering removes "bad" probes either without or with multiple perfect match hits along the genomic sequence and, (ii) it re-arranges "redundant" probe sets addressing the same gene, transcript or exon into one probe set. The resulting updated probe sets contain variable numbers of probes ranging from four to more than thirty. The mean probe set size is increased for gene- and transcript related sets (e.g., for the HG-U133A array: ENSEMBLE(gene)~14.9; ENSEMBLE(transcript)~13.9; Refsequ~14.9) and decreased for exon-related sets (ENSEMBLE(exon)~9.3) compared with the original Affymetrix set definition (NetAffx~11.1).

In Figure 9 and Table 3 we compare the hook characteristics for different probe set definitions. All updated probe set definitions under consideration give rise to very similar hook curves which essentially also agree with that obtained from the original probe set-assignments. This result again shows that the expressed probe sets follow the same hybridization law where changes of their performance will change their position along the hook. Interestingly, also the decay rates of the density distributions and the mean expression index <φ> of about 2.1 are very similar for all considered cases. This result indicates that the expression degrees of present probe sets located in the mix-, S- and sat-ranges of the original hooks remain, on the average, essentially unchanged after updating the probe sets.

The amelioration of the probe sets masks out a certain amount of "bad", i.e. falsely annotated or ambiguous probes and merges redundant probe sets (see above). As a consequence, the fraction of absent probe sets notably decreases from 34% (A-chip) and 50% (P-chip) to about 20% in both cases (see Table 3). The percentage of probe utilization inversely correlates with the reduction of the amount of absent probes detected by the hook method between the original and updated probe sets (see Table 3). For example, about 70% probes of the A-chip but only 50% of the P-chip are used after updating the gene-annotations. The obtained common percentage of absent probe sets of 20% reflects the consistent filtering criteria applied to both chip types. Indeed, the verification of probe sets based on genomic sequence data comes out with similar percentages of modified and not-modified probe sets sharing the same target in the original and updated probe set definitions.

In summary, the verification of probe sets increases the amount of the probe sets detected as present ones on one hand. Hence, the hook-calling criterion automatically removes the "bad" probe sets from further analysis. On the other hand, the mean expression degree and the hybridization characteristics reported by the ensemble of probes synthesized on the chip remain virtually unaffected by the redefinition step. Comparison of the updated expression measures of the slightly diverging probe sets shown in Figure 8 after verification leaves the small systematic biases essentially unchanged (data not shown).

## 4. Hybridization control

Assessment of data quality is an important component of the analysis pipeline for gene expression microarray experiments. Essentially all steps of RNA-preparation (extraction, amplification, in-vitro transcription, labelling), hybridization, washing and signal detection can have significant effects on the extracted "apparent" expression values seen between different samples with consequences for subsequent downstream applications. There are, for example, "technical" factors associated with the correction for background fluorescence owing to bleed over-effects from surrounding probes on the arrays [32], or to spatial artefacts [33,34]. Another kind of effects are linked with the RNA integrity and the used amplification and labelling protocols [35-39]. In this section we demonstrate the potential of the hook-analysis to detect and to estimate variations of the data owing to RNA-quality, the effect of substitution of cRNA by cDNA and of the labelling protocol.

### RNA-amplification bias

The amplification step of cRNA-preparation uses reverse transcriptase primers starting from the 3' -end of the original mRNA resulting in a population of 3' -biased, truncated transcript fragments. This 3'-overrepresentation gives rise to the systematic lowering of signal-intensities when the position of the probes shifts towards the 5'-end [35,40,41]. Hence, the probes designed for detecting one and the same transcript apparently report a progressively decreasing expression degree with increasing distance from the 3'-end of the transcripts. This is potentially detrimental for the expression value of the probe set summarized from individual probe-level data.

**Figure 10:**
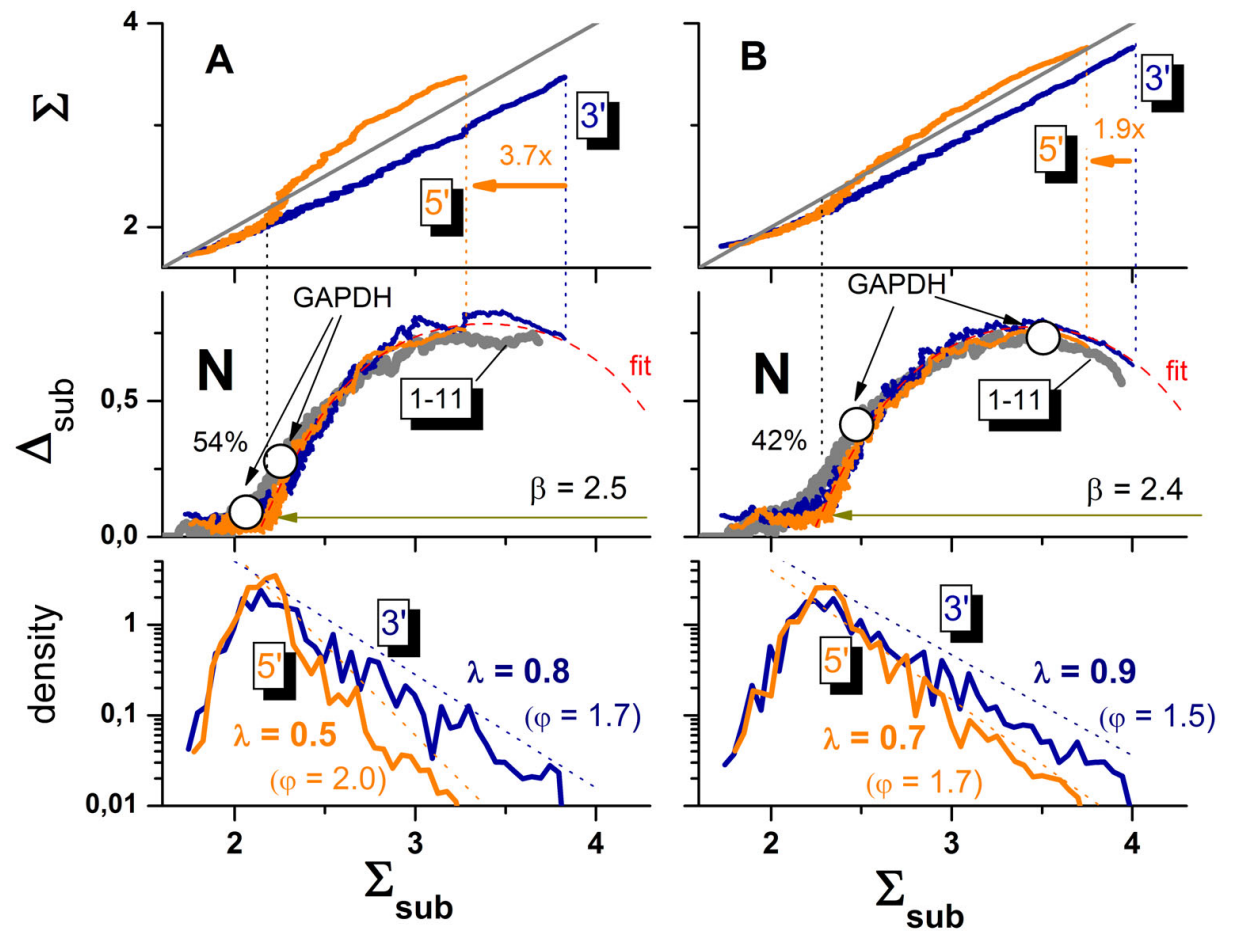
3'/5'-bias of two replicated hybridizations A (left part) and B (right part) of RNA of different quality on the rat genome array RAE-230: The graph above, in the middle and below show the total log-averaged mean of the PM- and MM-intensities, Σ, taken over all 11 probe-pairs of each set, the hook curves and the signal distributions, respectively, as a function of the sub-mean, Σ_sub_, averaged over subsets of the first four probes of a probe set (probes no. 1–4) closer to the 5'-end of the transcripts and the last four probes (no. 8–11) nearer to the 3'-end of the transcripts. The 5'- and 3'-biased sub-means virtually agree in the N-hybridization range whereas upon specific hybridization the 3'-biased sub-mean exceeds that of the 5'-biased one owing to 3'-biased amplification of RNA (upper panel, see arrows, the factors indicate the fold changes of the 3'-end relative to the 5'-end). The dimensions of the different hooks calculated using either all probes (1–11) or the biased subsets roughly agree each with another showing that all probes follow virtually the same hybridization law (middle panel). The higher yield for RNA fragments near the 3'-end of the transcripts gives rise to larger decay constants λ if one plots the signal-density as a function of the Σ_sub_-coordinate of the respective subsets of probes (see lower part, φ is the respective mean negative logarithmic expression index). The 3'/5'-bias is larger for sample A shown in the left column of the figure. Note that the width of the respective hooks and the fraction of absent probes (42% versus 54%, see figure) increase upon decreasing RNA-quality (compare A with B). The open circles in the middle panel indicate the positions of the GADPH-probe sets used typically for 3'/5'-hybridization control. The left one in each hook refers to the 5'-biased set and the right one to the 3'-biased set.

To illustrate the consequences of the 3'-biased amplification on the hook-data we ranked each probe in each probe set according to its position from the 3'-end, calculated the Δ- and Σ-coordinates as average value over probes no. #1 – #4 (subset more closely to the 5'-end), #8 – #11 (subset more closely to the 3'-end) and #1 – #11 (total probe set) and presented the hook-plots, the density distributions and the total Σ-coordinates as a function of the "sub-Σ-values", Σ_sub _in Figure 10. This approach considers the sequential ordering of the probes as a rough measure of their actual position along the respective gene to estimate the mean effect of the 3'-biased transcript populations on the hook-characteristics.

As an example, the figure compares two biological replicates A and B of total RNA prepared from rat muscle hybridized on rat genome RG-230 GeneChip arrays. Before microarray analysis RNA integrity and concentration was examined on an Agilent 2100 Bioanalyzer (Agilent Technologies, Palo Alto, CA, USA) using the RNA 6.000 LabChip Kit (Agilent Technologies) according to manufacturers instructions. Quantification of 28S and 18S ribosomal RNA before target amplification using the T7-protocol (see [42,36] and [43] and references cited therein) revealed virtually equal RNA quality from both preparations according to the 28S/18S-ratios of 1.45 (sample A) and 1.43 (B).

Figure 10 (upper part) correlates the total probe set average of the probe intensities, Σ, with that of the subsets, Σ_sub_. In the N-hybridization range both sub-averages agree each with another. This result is plausible because the 3'-bias due to incomplete amplification of full-length transcripts applies per definition only to specific hybridization: Non-specific transcripts are not-specified with respect to their position relative to the 3'-end and thus they on the average hybridize equally to all probes regardless of their relative position. Upon increasing mean intensity-values Σ_sub _and Σ, the curves however split into two branches starting with the onset of specific binding. The 3'-biased sub-average exceeds that of the 5'-biased one by a factor of 3.7 (sample A) and 1.9 (sample B) in the S-range. This difference indicates the more uniform amplification in sample B providing a higher yield for longer transcripts. Note that the observed onset of the split between the 3'- and 5'-branches well agrees with the position of the break of the respective hook curve (see vertical dotted line). This type of analysis thus once more confirms the chosen break-criterion to estimate the boundary between the N- and mix-hybridization ranges along the hook curve.

The total hook and the 3'- and 5'-"subhooks" of each sample are well described by the same theoretical function using a common set of parameters (see middle panel in Figure 10). To a good approximation, all probes obey the same hybridization law irrespective of their position relative to the 3'-end and irrespective of their amplification yield. The intensities of the probes near the 3'-end however cover a larger Σ-range compared with the respective 5'-biased subset. This effect is manifested by the larger decay constant λ of the signal distribution of the 3'-biased probes compared with that of the 5'-biased probes as illustrated in the lower part of Figure 10. The larger λ indicates the better (specific) signal to non-specific background (S/N)-ratio: the average specific signal level is larger in units of the detection limit which is roughly given by the non-specific background. The 3'-subset is also characterized by the larger values of the (negative-logarithmic) expression index φ. It reflects the larger average strength of specific binding owing to the larger fraction of specific full-length transcripts.

The smaller 3'/5'-ratio in the S-range, the smaller expression index and the larger decay constants, λ, of sample B compared with sample A reveal a generally larger fraction of specific transcripts due to more complete amplification and thus a better RNA-quality. The hook-analysis also reveals that the larger fraction of full length transcripts in sample B is accompanied by a slightly smaller width of the hook and a smaller fraction of absent probes (see middle panel of Figure 10). The latter trend can be simply attributed to the fact that the mean occupancy of the probes with specific transcripts and thus the specific signal increases if one improves the RNA-quality in terms of longer transcripts. Note that the increase of the decay constant upon RNA-improvement means that the probe sets on the average shift towards the S-range of the hook-curve. This trend is accompanied by a reduction of the percentage of absent probes from 54% to 42%. MAS5-analysis provides a similar difference of the amount of absent probes with 63% and 53% for the A- and B-samples, respectively.

The narrowing of the hook upon improvement of RNA quality, indicates a larger relative amount of non-specific binding. This trend seems peculiar because one might expect that the larger amount of specific transcripts reduces the amount of non-specific binding. However, the more efficient amplification step in sample B results in a higher total number of full length transcripts and/or in a larger binding constant for non-specific binding and thus in an increased binding strength of non-specific binding which, in turn, gives rise to the increased level of cross-hybridization as indicated by the slightly narrower hook-curve. Note however that the decreased quality of the RNA-amplification only weakly shifts the rising branch of the hook curve, in contrast to the overall dilution effect shown in Figure 5.

Microarrays of the GenChip-design contain special probe sets for estimating the 3'/5'-amplification bias. They refer to relatively long transcripts such as β-actin and GADPH with probe sets targeting the transcription of their 3'-, mid- (m), and 5'-regions. Small 3'/5'-signal ratios are generally thought to indicate small amplification bias and thus good amplification quality.

**Figure 11:**
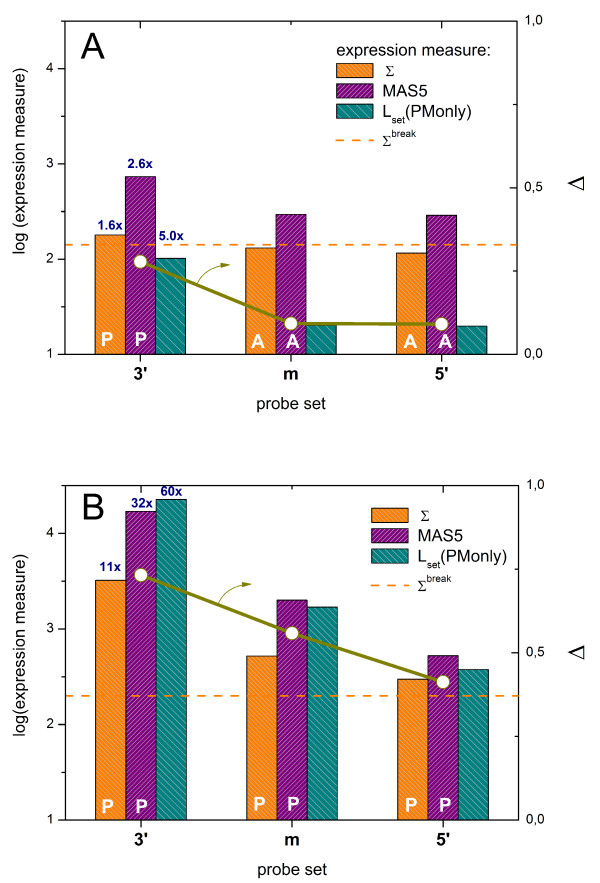
Characterization of the 3'/5'-amplification bias for the two samples shown in Figure 10 using the GADPH-probe sets Affx_rat_GADPH_x_at with x = 3', m and 5'. These three sets probe the GADPH-transcript with increasing distance from the 3'-end. The bars show the log-averages of the PM and MM intensities after correction for the optical background over the respective probe sets (Σ) and the MAS5 and hook (PMonly) expression estimates. The horizontal line indicates the hook-coordinate of the break, Σ^break ^with Σ ≥ Σ^break ^called present (P) and Σ < Σ^break ^called absent (A, see also the middle panel of Figure 10). Note that the Σ-signal of GADPH in sample A is dominated by non-specific hybridization at least for the m- and 5'-probes whereas it contains a much larger specific contribution in sample B. The fold-changes of the 3'/5'-signals are given above the 3'-bars. The circles indicate the respective Δ-coordinate of the hook-curve referring to the right axis.

Figure 11 compares the hook-coordinates (Σ and Δ) and two expression measures (MAS5 and hook/PMonly) of the three GADPH-probe sets in both samples. The intensity-related Σ-values of the different probe sets only marginally differ for sample A "pretending" this way better RNA-quality than for sample B with markedly larger 3'-values (see the fold changes above the bars: 1.6×-versus-11× for A and B, respectively). Comparison of the Σ-values with the break criterion however clearly indicates that the GADPH-signals of sample A are dominated by non-specific hybridization which was shown to level-out 3'/5'-expression differences (see also the open circles in the middle panel of Figure 10 which indicate the position of the 3'- and 5'-probe sets of GADPH along the hook curve). Note that both, the 5'- and m-sets of sample A are called absent by the hook method. Contrarily, all GADPH-sets are present in sample B. Their 3'/5'-ratio consequently can be attributed more reliably to the amplification bias whereas that of sample A simply reflects the virtual absence of GADPH-transcripts. Note that the expression values calculated by MAS5 and, to an even larger degree, hook (PMonly) reflect drastically increased 3'/5'-ratios owing to the N-background correction. Note also, that the 3'/5'-ratios of the GADPH-probe sets of sample B exceed that of the sub-hooks in the S-range (11×-versus-1.9×, see Figure 10). This difference simply reflects the longer transcript regions interrogated by the entire set of GADPH-probes compared with the mean transcript-length probed by the subhooks. Analysis of the alternative β-actin control set provides analogous results (data not shown).

In summary, the 3'/5'-ratio of the respective control probe sets are obviously insufficient for judging the amplification bias because non-specific hybridization keeps the signal of the 5' probe set at the same level as that of the 3' probe set which misleadingly pretends good amplification quality. Consideration of the hook-coordinates of these probes and, more reliably, analysis of 3'-biased "sub-hooks" enables the separation of the N- and S-hybridization ranges and this way a clear identification of the 3'/5'-amplification bias.

### Tissue specific RNA quality and normalization of microarray data

Measurement of gene expression is based on the assumption that an analyzed RNA sample closely represents the amount of transcripts *in vivo*. Transcripts show stability differences of up to several orders of magnitude raising the possibility that partial degradation during cell lysis and sample preparation causes a transcript-specific bias in the expression measures in addition to the amplification bias discussed in the previous section [37]. Different RNA quality measures, such as the 28S/18S ratio, the RNA integrity number (RIN) or a degradometer-score have been developed, verified (see [43] and references cited therein for an overview) and related to different microarray hybridization characteristics [36,39,42]. It was shown that the decrease in RNA integrity is often paralleled by the decrease of the percentage of present calls [37,39] which implies the reduction of the expression degree for degraded transcripts. Other studies however reveal more puzzling results, either with virtually no effects of degradation on expression or with opposite correlations between RNA-quality and weak and strong signals where the former ones increase and the latter signals decrease the worse the RNA becomes [38].

The integrity of the RNA extracted from different tissues systematically depends, among other factors, on the type of the tissue possibly and partly because of variations of the content and the activity of ribonucleases [37,39]. Estimation of RNA-quality and, if possible, appropriate correction for tissue-specific biases are thus essential steps in establishing tissue-specific expression profiles.

**Figure 12:**
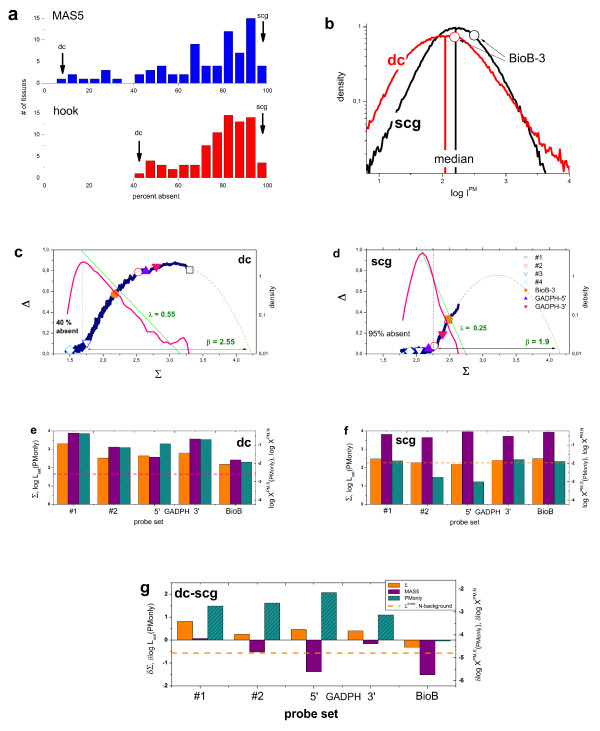
Tissue-specific RNA profiling. Part a) Frequency distribution of absent calls of tissue-specific total RNA hybridized on HG-U133A arrays taken from 79 tissues and analyzed with MAS5 and hook (raw array-intensities and MAS5 data were taken from ref. [44]). Part b) – g) Comparison of two hybridizations with small and large absent rates (see arrows in part a): peripheral blood-BDCA4 dentritic cells (dc, GEO-query GSM18873) and superior cervical ganglion (scg, GEO-query GSM19012). Both hybridizations used the same amount of total RNA (5 μg) for synthesis of biotinylated cRNA and the same labelling protocol. Part b) compares the log-intensity-distributions of the PM-probes: Except the shift and widening of the distribution of the dc-sample, one observes essentially no peculiar differences between the specimens. The median and the probe-set related values of BioB-3 are explicitly shown and discussed in the text. Parts c) and d) show the respective hook-plots together with the signal-density distributions. Note the striking differences: The scg-sample hybridizes much weaker with a markedly larger fraction of probe set with absent calls (95%) and a much steeper decay of the distribution in the mix-range of the hook (λ is the decay constant). The dotted curves are fits of the Langmuir model. The open symbols indicate the hook-coordinates of selected probe set in both preparations to illustrate the apparent expression changes from different regions of the hook (#1 to #4). The solid symbols refer to amplification and hybridization control probe sets. In part e) and f) the Σ-coordinates and the expression measures of these selected probes are explicitly shown, part g shows the respective log-differences between both samples. Note that the difference of the non-specific background level is negative (dashed horizontal line), whereas the difference of the specific binding strengths of most of the considered probe sets is positive (PMonly measures). The specific expression of the BioB-control is virtually invariant in both samples, as expected. Contrarily, MAS5 pretends significant expression changes of the BioB-control due to improper normalization (see text).

In Figure 12 we compare the hybridization characteristics of different tissues. The raw array data are taken from the comparative expression study on 79 human tissues [44]. All hybridizations use the same start-amount of 5 μg of total RNA and the same amplification, hybridization and labelling protocols. Part a of Figure 12 shows the distributions of the amount of absent calls obtained using MAS5 and hook-method for all considered tissues. The possible percentage of absent probe sets widely varies from values greater than 95% (virtually no present genes) to ~40% (hook) and ~10% (MAS5). Except their different spread, both distributions show essentially the same structure which reflects strong correlations between the MAS5 and hook calls in agreement with our previous findings (see above).

For more detailed analysis we select two samples with relatively large and small percentages of absent probe sets, the RNA of which were extracted from superior cervical ganglion cells (scg) and from periphal blood/dentritic cells (dc) (see arrows in Figure 12a), respectively. Comparison of the respective intensity distributions indicates, except the slightly divergent width, no striking differences (see Figure 12b). In contrast, the respective hook-plots and underlying signal-distributions shown in part c and d of Figure 12 reveal completely different hybridization characteristics: Most of the probe sets of the scg-hybridization accumulate within a relatively narrow Σ-range corresponding mainly to the N- and partly to the mix-hybridization regimes whereas the probes sets of the dc-sample cover a much wider range which includes the S- and sat-hybridization regimes as well.

The different shapes of the hook curves cannot be explained by a smaller amount of RNA (e.g. due to a smaller yield of cRNA synthesis), less-efficient labelling and/or suboptimal calibration of the scanner. In these cases one expects the shift of the "whole" hooks without considerable change of their width and decay of the density distribution (compare, e.g. with Figure 5, upper part). Instead, the hook of the scg-sample is distinctly reduced in width reflecting the much higher level of non-specific background hybridization paralleled by the reduction of the decay constant.

The hook-coordinates of selected probe sets are highlighted by symbols in Figure 12 to illustrate this result: The symbols refer to probe sets selected to cover essentially the N-, mix-and S-hybridization regimes of the dc-hook. In the scg-hook most of these sets shift towards, and partly behind the detection limit given by the break-criterion. The solid symbols refer to amplification (GADPH-3' and -5') and hybridization (BioB-3) controls. The horizontal shift between amplification controls (see the solid triangles, the left one refers to the 5'- and the right one to the 3'-probe set) suggests a slightly smaller amplification bias of the dc-sample. The transcripts for hybridization controls (the solid circle refers to BioB_3) were added to the RNA-extracts in constant amounts before the inverse transcription step to assess its performance. The position of the respective Σ- and Δ-coordinates along the hook-curve remains relatively invariant indicating that the inverse transcription step has been performed in both samples in comparable quality. The drastic differences in the call rates must be therefore attributed to tissue-specific differences of the RNA-quality.

In parts e and f of Figure 12 we show the Σ-coordinates and the expression measures of the selected probe sets in both samples. Part g provides the differences (dc – scg) between them. The log-intensity measures (Σ) and the PMonly hook-expression values clearly reveal the larger signal and expression level of the dc-sample. Importantly, the PMonly-expression estimate of the BioB-hybridization control remains invariant between the samples. This result correctly reflects the equal amounts of BioB-transcripts spiked into both samples. The difference of the Σ-coordinates is however negative for BioB-3. This result and the fact that the differential expression of the PMonly estimates exceeds that of the Σ-data can be attributed to the non-specific background contributing to the latter data. The larger N-background in the scg-sample effectively increases the respective signal. Moreover, the data clearly show that the positive difference of the log-binding strength of specific hybridization of most of the transcripts is counterbalanced by the negative change of the binding strength of non-specific binding (see the horizontal dashed line in part e) – g) of Figure 12).

These trends partly explain the puzzling results of a recent correlation analysis between signal intensities and the degree of RNA-degradation [38]: Our data show, that, on one hand, degradation of RNA increases the non-specific background level with the consequence that the intensities of probes with small specific signal contributions effectively increase. On the other hand, the specific binding strength decreases upon RNA-degradation with the consequence that the signals of strongly expressed signals decrease. The former effect mainly affects weak intensities whereas the latter effect is more relevant for stronger total signals. Both opposite effects contribute to the intensity of each probe with specific weights giving rise to increased, decreased or even unchanged total signals.

In part e – g of Figure 12 we also show MAS5 expression estimates taken from ref. [44]. The MAS5-expression measures of the dc-sample agree to a good approximation with that of the hook-method (see Figure 12, part e). For the scg-sample MAS5 however provides a considerably larger mean expression level. As a result, the expression differences are either much smaller in magnitude, or more critically, even change sign compared with the hook-results (see part g of Figure 12: dc-scg). For example, the hybridization control BioB becomes apparently much less expressed in the dc-sample in contrast to the hook-method which detects essentially no change, as expected.

These qualitative discrepancies between both approaches uncover a fundamental problem of microarray normalization with no satisfactory solution yet (see, e.g., [45]). Note that in their analysis the authors used MAS5 together with global median normalization of the raw intensities [44]. The vertical bars in part b of Figure 12 indicate the median of the log-intensity distributions. For the two considered samples the change of the non-specific contribution clearly dominates the observed change of the median chip intensity resulting in a stronger median signal of the scg-sample. The relative effect of, e.g. BioB with respect to the median is larger for the scg-sample (see the open circles in the figure) which gives rise to the negative differential expression reported by MAS5. This result exemplifies the problem with normalization methods which rescale the individual chip intensities to global chip characteristics such as their median or average value or use an averaged distribution as by quantile normalization. For the particular example discussed here such methods mask the larger specific signal in the dc-sample. Contrarily, the hook method disentangles the specific and non-specific signal-contributions with the option to scale them separately in subsequent normalization steps.

### Labelling protocol

**Figure 13:**
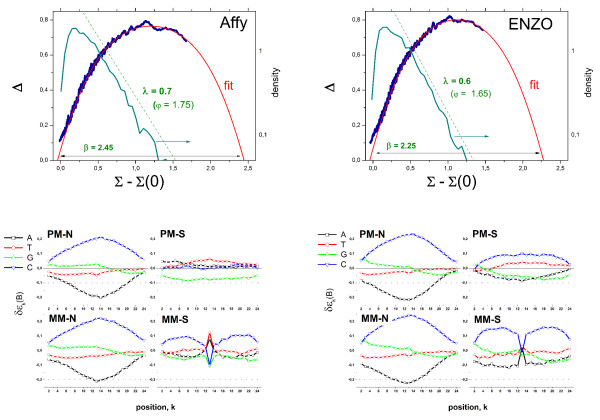
Hook analysis of two replicated hybridization on RAE-230 rat genome arrays which are labelled using either the Affymetrix- (left) or ENZO- (right) protocols. The Affy-protocols labels the cytosines only whereas the ENZO-protocol labels cytosines and uracyls as well.

In addition to the quality of start-RNA and the amplification bias there are other methodological differences such as the labelling reaction that can introduce systematic biases. Figure 13 compares the hook characteristics of two replicated samples of the same amount of starting RNA (5 μg) which are labelled using two different in-vitro-transcription (IVT) labelling kits: the Enzo BioArray high-yield RNA transcript labeling kit (Enzo) and the GeneChip expression 3'-amplification kit for IVT labeling (Affy) [8,46]. Both methods essentially follow the same experimental steps. Major distinction exists in the use of Biotin-UTP and -CTP in the former and Biotin-UTP only in the latter method. Fluorescent labels thus attach either to C- and U-nucleotides as well or to U-nucleotides only.

The sensitivity profiles of the N-hybridization range are very similar for both labelling protocols with differences of less than 20% of the respective sensitivity value. Similar results were reported previously by using either Biotin-UTP or Biotin-CTP [47]. The sensitivity terms additively decompose into "binding-"contributions related to the effective free energy of the respective base pairing; and into a fluorescence contribution taking into account base-specific labelling [20]. Labelling is expected to decrease the binding contribution (because the bulky label disturbs the base-base interactions) and to increase the fluorescence contribution [19,20]. The obtained positional dependent sensitivity profiles reveal that, if at all, labelling has only little effect.

On the other hand, the width of the hook curve and the decay constant of the density distribution for the Affy-protocol slightly exceed the respective values for the Enzo-labelling at identical percentages of absent probes (~33%) and at identical optical background levels in both preparations. The observed differences indicate the slightly smaller amount of non-specific binding and the stronger specific binding of the former preparation. Hence, the Affy-protocol slightly better performs then the previous Enzo-labeling because it reduces the non-specific background level and increases the effective binding strength for specific binding; this way, giving rise to both, a better specificity and sensitivity of the method [26] in agreement with the results of special benchmark experiments [46].

The molecular origin of the observed differences is presently not clear and requires further analyses. Note however that the Enzo-protocol introduces a significantly higher fraction of biotinylated nucleotides with potentially deteriorated binding affinities which provides a tentative explanation of the observed trends. The stronger specific binding caused by the Affy-protocol is paralleled by stronger saturation effects at high intensities which, in turn, give rise to systematic differences between the S-sensitivity profiles of both preparations: The profiles of cytosine (C) and guanine (G) shift systematically towards smaller sensitivities whereas the T- and especially the A-profiles shift into the opposite direction. This vertical "compression" of the profiles was previously observed [20]. It reflects the fact that stronger base Watson-Crick pairings of the C- and G-nucleotides are, on the average over all probes, more affected by saturation than pairing of the T and especially A which form weaker bonds. Note also that the saturation effect is much smaller for the MM as expected. These results reveal that the hook-algorithm only incompletely corrects the individual probe intensities for saturation effects probably because the intensity asymptote upon complete saturation is not a chip constant but a sequence- and thus probe-specific property owing to washing effects [29,48].

### Replacing RNA targets with DNA

**Figure 14:**
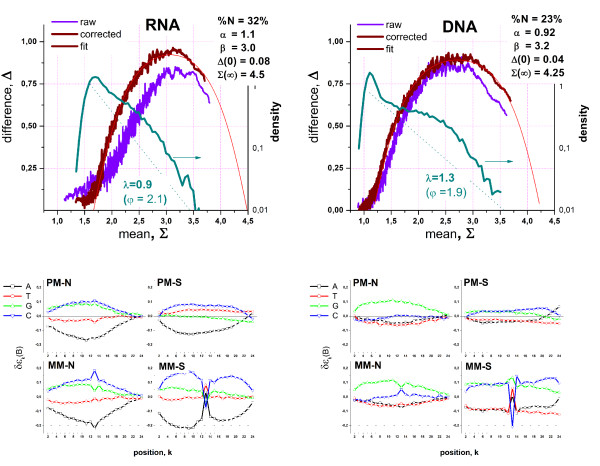
Hook-characteristics of cRNA (left) and cDNA (right) hybridizations prepared from of a Jurkat-cell RNA-extract on HG-U133Av2 chips (raw data are taken from ref. [49]).

Microarray technology takes advantage of either of two types of chemical entities as the labelled target, RNA or DNA, considered to be virtually equivalent for the purpose of expression analysis. RNA is usually hybridized on "conventional" expression arrays whereas especially newer GeneChip generations such as exon- and tiling-expression arrays as well as genomic SNP- and re-sequencing-arrays use DNA-targets. Figure 14 compares selected hook characteristics of both options to illustrate the effect of the two binding "chemistries" using the same start RNA-extract prepared from Jurkat-cells (chip data are taken from [49]). cRNA was prepared by standard one round in-vitro transcription (see above) whereas cDNA was obtained by means of a different protocol (see [49] and references cited therein). Besides the chemical entity of the targets both protocols differ with respect to preparation steps such as fragmentation (chemical versus enzymatic), labelling ("during isothermal amplification" versus "after fragmentation") and the position of the label (throughout the sequence versus end-labelled).

Inspection of the hook-curves reveals several effects caused by the substitution of RNA by DNA: Firstly, the sensitivity correction to a much less extent affects the hook-curve of the DNA-hybridization (compare the corrected and raw hooks). For example, the width of the N-range of the raw RNA-hook (ΔΣ(N) ≈ 0.7) considerably exceeds that of the respective DNA-hook (ΔΣ(N) ≈ 0.3) whereas after correction the N-widths shrink to virtually identical values in both cases (ΔΣ(N) ≈ 0.2). Secondly, DNA/DNA hybridisation shifts the whole hook, and especially the background level, to smaller abscissa values indicating a smaller mean intensity level; thirdly, substitution of RNA by DNA slightly increases the width of the hook (β) and the decay constant of the density distribution in the S-range (λ); and fourthly, it slightly reduces the vertical dimension of the hook (α). Moreover, also the sensitivity profiles indicate characteristic differences: Especially, the profiles for Guanine (G) provide the largest contributions for DNA-binding to DNA-probes whereas the Cytosine-profiles are the largest in most cases for RNA-binding.

The different target-entities give rise to D(NA)/R(NA)- and D/D-base pairings in the target/probe-duplexes and to R/R- and D/D-interactions for bulk duplexing of the targets in solution. The thermodynamic stability of specific 27 meric oligomer-duplexes was found to follow the order D/D < D/R < R/R with free energy ratios (37°C) of ΔG(D/D)/ΔG(D/R) ≈ 0.9 and ΔG(R/R)/ΔG(D/R) ≈ 1.3 [50]. Note that the PM/MM-gain α ≈ log(s) approximately refers to the free energy increment of one Watson-Crick pairing in 25 meric probe/target duplexes if one neglects the specific mismatch contribution. The decreased PM/MM-gain (α) of the DNA-hybridization thus corresponds to the weaker association of D/D -versus – D/R where the ratio α (D/D)/α (D/R) ≈ 0.85 ± 0.05 roughly agrees with the expected free energy ratio.

The slightly larger width of the DNA-hook indicates the smaller non-specific binding strength of the D/D-duplexes. This difference and the larger variability of the RNA-hybridization were attributed to relatively-stable, mismatched "G•u-wobble" base pairings in the non-specific R/D-duplexes (the lower case letter refers to the target, the upper case letter to the probe) which give rise to less specific binding and stronger scattering of the background compared with D/D hybridizations without such relatively-stable mismatched pairings [49]. The latter D/D-hybridization is consequently more specific than the R/D-hybridization as indicated by the larger decay constant (see Figure 14) [26].

Also the sensitivity profiles indicate systematic differences of base-pair interactions in both hybridizations. Particularly, the relative values of the G- and A-profiles for the D/D-duplexes are considerably larger than that for the D/R-duplexes. Exactly this trend is expected from the relative interaction strength of canonical Watson-Crick pairings in the respective duplexes: D/D-pairings are symmetrical with respect to "bond-reversals" (i.e. C•g≈ G•c > A•t≈ T•a) in contrast to "unsymmetrical" D/R-interactions (C•g > G•c≈ T•a > A•u) [19,20,50-52]. Hence, for D/D-duplexes one expects the relative enhancement of the G and A sensitivity terms compared with those in the D/R duplexes in agreement with the observed profiles.

Note however that the sensitivity profiles refer to effective binding strengths which include surface and bulk interactions as well [26,53]. Such effects give rise to specific differences between the S- and N-profiles especially of the RNA-preparation which implies relative strong R/R-interactions in the respective bulk duplexes.

## 4. Summary and Conclusion

We presented a new method of microarray data analysis based on a physical model. This so-called hook method pre-processes the raw intensity data for further downstream analyses on one hand, and, on the other hand, provides chip characteristics with potential applications in hybridization quality control and array normalization.

In this publication we illustrate the diagnostic potential of the hook-method by means of different chip- and transcript-related characteristics in various situations:

- Using the data of spiked-in and dilution experiments it was shown that our single-chip approach provides accurate and precise expression measures over three orders of magnitude in units of the specific binding strength of the transcripts. The correction for saturation and probe-specific non-specific background assures linearity between the input (transcript concentration) and output (expression degree) measures. Among the four alternative measures, PMonly and PM-MM-difference measures perform best, but also the measure extracted from the S/N-ratio provides satisfactory results.

- The "present/absent"-concept of detection calls originally introduced by Affymetrix provides straightforward, simple and helpful information which relates the signal of each transcript to the detection limit of the particular hybridization and, in addition characterizes the mean "presence" of transcripts in the hybridization solution. The hook-method calculates an analogous measure based on the break-criterion reflecting the onset of specific hybridization. This criterion implicitly takes into account the different correlations between the PM and MM probes upon non-specific and specific hybridization and thus it "dynamically" adapts to each particular hybridization. We have shown that this criterion well classifies into present and absent transcripts using data taken from the two-species yeast 2.0 array and from the golden spike experiment with known batches of "empty" probes.

- The hook method performs reasonably well by comparing expression data of the same origin between two chip generations (HG-U133A and HG-U133 plus 2.0). The hook-diagnosis suggests that subtle differences of the hybridization law due to details of chip-manufacturing and/or -handling upon preparation give rise to slightly biased expression data between different array types and/or different batches of chips of the same type.

- The re-assembly and filtering of probe sets based on improved genomic information increases the amount of probe sets detected as present ones. This result in turn shows that the hook-calling criterion applied to the original probe set definitions partly removes the "bad" (because of inconsistent probe assignments) probe sets from further analysis. The mean hybridization characteristics remain virtually unaffected by the redefinition step of the probe sets. The consequences of probe set-updating for the expression measures on transcript level will be studied separately.

- The effect of 3'-biased RNA amplification gives rise to the progressively decreased specific hybridization of probes with increasing distance of their position relative to the 3'-end of the transcript which can be detected by hook-analysis using appropriate subsets of probes nearer to the 3'- and the 5'-end, respectively. This analysis properly differentiates between specific and non-specific hybridization where the latter one is, per definition, not affected by the 3'-biased intensity effect. Our data show that overall 3'/5'-signal ratios not considering the difference between specific and non-specific binding can lead to misinterpretations of the amplification bias.

- Hook analysis reveals detailed insights into consequences of tissue-specific RNA-quality differences on hybridization and expression measures. Degradation of RNA increases the fraction of absent probes paralleled by the decrease of the specific binding strength and counterbalanced by the increase of non-specific background hybridization. Improper separation of both opposite effects can pretend expression changes into the wrong direction. We suggest that the chip characteristics provided by the hook method can serve as calibration benchmarks for alternative normalization algorithms which take into account the different behaviour of the specific and non-specific signal in samples of varying RNA-quality.

- The variation of the labelling protocol and substitution of RNA-targets by DNA modifies the probe/target interactions. Hook analysis shows for example that DNA-targets, and to a smaller degree, the Affy-labeling protocol (no labelling of cytosines) improve the specificity of the method compared with RNA-targets and the previous ENZO-protocol, respectively. For DNA-targets the sequence correction is of much smaller impact because of smaller sequence-induced variability of the raw intensities.

In summary, sequence correction and especially the quantification of the non-specific background contribution for each probe enable subtle diagnosis of the hybridization on each array. To extract this information the hook method combines the intensities of each PM/MM-probe pair and utilizes the different properties of both probe types. Here the MM behave like "weak-affine" PM and serve as intrinsic reference for the PM over the whole potential concentration range of the transcripts. We illustrated that this intrinsic referencing might be extremely useful for dealing with practical issues of expression analysis such as RNA-quality, hybridization control and calibration of expression measures. This publication outlined several potential applications of the method which will be addressed in our future work.

## Competing interests

The authors declare that they have no competing interests.

## Authors' contributions

HB designed and leads the project, carried out most of the analyses and wrote the paper. SP wrote the computer program for hook analysis and helped to draft the paper. KK added experimental expertise and helped to draft the paper. All authors read and approved the final manuscript.
